# Genome Sequences of Four Clinical *Staphylococcus aureus* Strains with Diverse Drug Resistance Profiles Isolated from Diabetic Foot Ulcers

**DOI:** 10.1128/genomeA.00204-14

**Published:** 2014-03-20

**Authors:** Thokur Sreepathy Murali, Bobby Paul, Hersh Parikh, Rana Pratap Singh, Shettigar Kavitha, Manoj K. Bhat, Kapaettu Satyamoorthy

**Affiliations:** a Department of Biotechnology, School of Life Sciences, Manipal University, Manipal, India; b Invitrogen Bioservices Pvt., Ltd., Gurgaon, India

## Abstract

*Staphylococcus aureus* is a major pathogen associated with diabetic foot ulcer infections. To gain insight into their pathogenicity and virulence potential, we report draft genome sequences of four strains of *Staphylococcus aureus*, isolated from diabetic foot ulcers, showing profiles with various degrees of resistance to common antibiotics.

## GENOME ANNOUNCEMENT

Infections in diabetic foot ulcers are among the major comorbid conditions leading to mortality in diabetic individuals (1, 2). To elucidate the pathogenic potential of bacterial communities associated with infected diabetic foot ulcers, we carried out culture-dependent isolation of bacteria from infected wound beds. Samples were obtained from patients visiting a tertiary hospital, Kasturba Medical College, Manipal, India, and a total of 357 diabetic ulcer samples were collected. From these samples, we isolated 940 bacterial strains, and these were further analyzed using traditional and molecular techniques to assess the diversity. Though an abundance of Gram-negative bacilli and Gram-positive cocci were obtained, *Staphylococcus aureus* was frequently isolated from the wound samples. *Staphylococcus aureus* is considered the major pathogen associated with diabetic foot ulcers and is also one of the most adaptable human pathogens, known to cause a wide range of diseases (3). Though worldwide methicillin-resistant *Staphylococcus aureus* (MRSA) strains are a major cause of hospital-acquired infections, there are also reports of emergence of MRSA and methicillin-susceptible *S*. *aureus* (MSSA) from other environmental settings (4). Extensive structural features, including specific surface components that aid in adhesion to host tissues and an array of secreted proteins/toxins expressed by *S. aureus* strains, play major roles in enhancing the virulence potential of these organisms (5, 6). The strains obtained were tested for their sensitivity to amikacin, ampicillin, amoxyclav, ciprofloxacin, chloramphenicol, erythromycin, gentamicin, linezolid, vancomycin, and cefoxitin. We selected four strains with profiles showing various degrees of resistance (resistance to one to nine antibiotics tested) to the above-listed antibiotics for whole-genome sequencing, and the draft genome sequences are reported here. Genomic DNAs from the *S. aureus* strains were isolated using the phenol-chloroform method, and whole-genome sequencing was performed with the Ion Torrent Personal Genome Machine (Life Technologies, CA) per the manufacturer’s guidelines. A total of 1,624,573 to 2,469,063 reads were obtained, with coverages of 59.3× to 83.97× (Table 1). *De novo* assembly was performed using the MIRA-4 assembler (7), which yielded 59, 58, 125, and 74 contigs for *Staphylococcus aureus* strain MUF168, *S.* aureus strain MUF256, *S. aureus* strain MUF270, and *S. aureus* strain MUF475, respectively. The GC contents for the four strains ranged from 32.66 to 32.78%. The functional annotation of the assembled genomes was performed by the Prokaryotic Genome Annotation Pipeline v2.0 (NCBI) for deposition with the Genome database. A total of 2,433 to 2,728 coding sequences (CDS) were predicted for these four strains (Table 1). All four of the genomes harbored genes coding for beta-lactamase, acyl carrier protein, chloramphenicol resistance, teicoplanin resistance-associated membrane protein TcaB, fibronectin binding proteins A and B, polysaccharide intracellular adhesin biosynthesis proteins, siderophore staphylobactin biosynthesis proteins, staphostatin A, staphylocoagulase, and staphylokinase.

**Table 1:** *Staphylococcus aureus* genome assembly and annotation statistics

Strain	No. of contigs	Fold coverage	*N*_50_ (bp)	Consensus length (bp)	GC content (%)	No. of CDS	Accession no.
MUF168	59	67.67	96,843	2,752,244	32.78	2,433	AZQR00000000
MUF256	58	69.54	87,410	2,783,966	32.66	2,433	AZSE00000000
MUM270	125	83.97	52,369	2,834,436	32.76	2,728	AZSF00000000
MUM475	74	59.3	128,108	2,852,107	32.67	2,620	AZSG00000000

### Nucleotide sequence accession numbers.

The draft genome sequences have been deposited at GenBank under the accession numbers AZQR00000000, AZSE00000000, AZSF00000000, and AZSG00000000.
